# The proteasome activator REGγ accelerates cardiac hypertrophy by declining PP2Acα–SOD2 pathway

**DOI:** 10.1038/s41418-020-0554-8

**Published:** 2020-05-18

**Authors:** Yifan Xie, Yang Gao, Rifeng Gao, Wenlong Yang, Zheng Dong, Robb E. Moses, Aijun Sun, Xiaotao Li, Junbo Ge

**Affiliations:** 1 Department of Cardiology, Zhongshan Hospital, Fudan University, 180 Fenglin Road, Shanghai, 200032 China; 2 Institutes of Biomedical Science, Fudan University, 180 Fenglin Road, Shanghai, 200032 China; 3 Shanghai Institute of Cardiovascular Diseases, 180 Fenglin Road, Shanghai, 200032 China; 4 Department of Molecular and Cellular Biology, Baylor College of Medicine, One Baylor Plaza, Houston, TX 77030 USA; 5 Shanghai Key Laboratory of Regulatory Biology, Institute of Biomedical Sciences, School of Life Sciences, East China Normal University, 500 Dongchuan Road, Shanghai, 200241 China

**Keywords:** Proteasome, Cardiomyopathies

## Abstract

Pathological cardiac hypertrophy eventually leads to heart failure without adequate treatment. REGγ is emerging as 11S proteasome activator of 20S proteasome to promote the degradation of cellular proteins in a ubiquitin- and ATP-independent manner. Here, we found that REGγ was significantly upregulated in the transverse aortic constriction (TAC)-induced hypertrophic hearts and angiotensin II (Ang II)-treated cardiomyocytes. REGγ deficiency ameliorated pressure overload-induced cardiac hypertrophy were associated with inhibition of cardiac reactive oxygen species (ROS) accumulation and suppression of protein phosphatase 2A catalytic subunit α (PP2Acα) decay. Mechanistically, REGγ interacted with and targeted PP2Acα for degradation directly, thereby leading to increase of phosphorylation levels and nuclear export of Forkhead box protein O (FoxO) 3a and subsequent of SOD2 decline, ROS accumulation, and cardiac hypertrophy. Introducing exogenous PP2Acα or SOD2 to human cardiomyocytes significantly rescued the REGγ-mediated ROS accumulation of Ang II stimulation in vitro. Furthermore, treatment with superoxide dismutase mimetic, MnTBAP prevented cardiac ROS production and hypertrophy features that REGγ caused in vivo, thereby establishing a REGγ–PP2Acα–FoxO3a–SOD2 pathway in cardiac oxidative stress and hypertrophy, indicates modulating the REGγ-proteasome activity may be a potential therapeutic approach in cardiac hypertrophy-associated disorders.

## Introduction

Cardiac hypertrophy is an important adaptive response to pathological stimuli, but prolonged hypertrophy resulting in cardiac dysfunction and heart failure (HF) [1].

The proteasome system and lysosomal-autophagy pathway are the two most important intracellular mechanisms for regulatory protein degradation [2–5].

The 26S proteasome holoenzyme consists of a 20S proteolytic core and a regulatory particle that is required for its activation. The 19S regulatory particle is the classical and well-studied, which is composed of 17 subunits (PSMC1–6; PSMD1–4, 6–8, and 11–14) [6], activates 20S proteolytic core in an ATP- and ubiquitin-dependent manner. Three proteasome regulators, PA28/11S (PSME1-3), PA200/Blm10 (PSME4), and ECM29, were found to compete with the 19S regulatory particle, in an ATP- and ubiquitin-independent manner [7]. Dysfunction of proteasome activity causes a number of cardiac proteinopathies and eventually lead to HF [8–11].

REGγ (also known as PA28γ, PSME3) is a member of the11S family (REGα, β and γ) of proteasome activators “caps” that have been shown to bind to and activate the 20S core proteasome. It has been shown to promote the degradation of several important cellular regulatory proteins in a ubiquitin- and ATP-independent manner, including SRC-3, p21, p16, p19, p53, PKAcα, CK1δ, SirT1, ikBε, GSK3β, SirT7, KLF2, c-Myc, and Smad7, among others [12–15], in the regulation of a broad range of physiological and pathological processes, including cancer progression [16–19], development [20], premature aging [21], hepatic lipid and energy metabolism [22, 23], angiogenesis and atherogenesis [24, 25], oxidative response [26], bacterial infection [27], innate immunity and inflammatory diseases [28–30], and cardiac viral infection [15, 31], but the function of REGγ in protein quality control of cardiac hypertrophy is unclear.

In this study, we found that REGγ was significantly upregulated in the transverse aortic constriction (TAC)-induced hypertrophic hearts and depletion of REGγ in mice after TAC operation results in a massive inhibition of reactive oxygen species (ROS) accumulation and protein phosphatase 2A catalytic subunit α (PP2Acα) decay in heart and ameliorates pathological cardiac hypertrophy in a PP2Acα–FoxO3a–SOD2-dependent manner.

## Materials and methods

### Animal models

REGγ−/− mice with C57BL/6 genetic background were acquired from John J. Monaco (University of Cincinnati College of Medicine, Cincinnati) [30]. Mice were bred in the Animal Core Facility by following the procedures approved by the Baylor College of Medicine Institutional Animal Care and Use Committee. To generate the animals required in this study, we maintained REGγ+/− mice and kept intercrosses between males and females for more than six generations. Genotyping of REGγ+/+ and REGγ−/− mice was carried out by PCR analysis of genomic DNA as described [30]. Cardiac hypertrophy was induced in 8-week-old mice by TAC operation for 4 weeks. For manganese 5, 10, 15, 20-tetrakis-(4-benzoic acid) porphyrin (MnTBAP) treatment, MnTBAP was dissolved in PBS, and given to mice at a dose of 5 mg/kg/day for 4 weeks. Echocardiography was performed using a VisualSonics Vevo770 ultrasound biomicroscope (VisualSonics Inc, Toronto, ON, Canada) with a 15-MHz linear array ultrasound transducer. The surgeon or investigator was blinded to the mouse groups or genotypes. The left ventricular (LV) was assessed in both the parasternal long-axis and short-axis views at a frame rate of 120 Hz. End-systole or end-diastole was defined as the phase obtained with the smallest or largest LV area, respectively. LV end-systolic diameter (LVESD) and LV end-diastolic diameter (LVEDD) were measured from the LV M-mode tracing with a sweep speed of 50 mm/s at the papillary muscle level. LV fractional shortening (FS) were calculated by ([LVEDD−LVESD]/LVEDD).

### Cell culture, expression constructs, and reagents

Human cardiomyocytes AC16, rat cardiomyocytes H9C2, and 293T cells were purchased from ATCC and cultured in Dulbecco’s modified Eagle’s medium (DMEM) (Invitrogen, Carlsbad, CA, USA) containing 10% fetal bovine serum (FBS) (Invitrogen). The REGγ, PP2Acα, FoxO3a, SOD2, and p21 plasmids were constructed into pcDNA3.1 with Flag/HA/GFP-tag at the N terminus by proof-reading PCR and verified by sequencing. Angiotensin II (Ang II) was obtained from Sigma (Burlington, MA, USA). Dimethyl sulfoxide, okadaic acid (OA), cycloheximide (CHX), and MG132 were obtained from Sigma, and 4′,6-diamidino-2-phenylindole and dihydroethidium (DHE) were obtained from Invitrogen, and MnTBAP was obtained from Merck (Darmstadt, Germany).The following antibodies were used in this study: anti-β-actin antibody (A2228#, Sigma), anti-Flag/HA/GFP antibody (14793#/3724#/2956#, Cell Signaling, Danvers, MA, USA), anti-REGγ antibody (ab157157#, Abcam, Cambridge, UK), anti-SOD2 antibody (13141#, Cell Signaling), anti-PP2Acα antibody (ab137825#, Abcam)/(Proteintech), anti-phosphorylated FoxO3a antibody (ab26649#, Abcam)/(Cell Signaling), anti-ANP antibody (ab225844#, Abcam), anti-p21 (2947#, Cell Signaling), and anti-immunoglobulin G (IgG) (3900#, Cell Signaling). Real-time quantitative-polymerase chain reaction (RT-qPCR) was performed using a real-time PCR kit (Takara, Kusatsu, Shiga, Japan). Immunoprecipitation was performed using FLAG-M2 agarose beads (Sigma) or Pierce™ Protein A/G Agarose beads (Thermo Fisher, Waltham, MA, USA) and cell fractionation assay was performed using Nuclear and Cytoplasmic Extraction Kit (Thermo Fisher). Luciferase assay was performed using a luciferase assay system (Promega, Madison, WI, USA). In vitro protein degradation assay was performed using purified recombinant REGγ protein (R&D systems, Minneapolis, MN, USA) and 20S proteasome (R&D systems), and TNT^®^ Transcription/Translation System (Promega).

### Primary culture of neonatal rat cardiomyocytes (NRCMs)

The hearts from 1–3-day-old SD rats were cut into approximately nine to ten pieces and dissociated with 0.04% trypsin and 0.07% type II collagenase. After dispersed cells were incubated on 100-mm culture dishes for 90 min at 37 °C in 5% CO2, nonattached cells were collected and transferred into six-well plates, which were previously treated with laminin (10 μg/mL), and then 0.1 M 5-bromo-2ʹ-deoxyuridine was added. Primary cardiomyocytes were incubated in DMEM/F12 (Invitrogen) supplemented with 10% FBS for 16 h and then replaced with serum-free DMEM/F12, which contained appropriate chemicals.

### Histological analysis

For histological analysis, hearts were arrested with a 10% potassium chloride solution at end-diastole and then fixed in 4% paraformaldehyde. Fixed hearts were embedded in paraffin and cut transversely into 5 μm sections. Serial heart sections were stained with hematoxylin and eosin (H&E) or wheat germ agglutinin (WGA) (Invitrogen) to measure myocyte cross-sectional areas or masson staining to measure cardiac fibrotic areas or immunohistochemistry was performed as described [17] to show indicated protein expression in heart tissues. The degree of cardiac collagen deposition was detected by masson staining, and images were analyzed using a quantitative digital image analysis system (Image-Pro Plus 6.0).

### Transmission electron microscopy (TEM)

LV tissues were dissected into small cubic pieces ≤1 mm^3^ and fixed with 2.5% glutaraldehyde (pH 7.4) for more than 2 h. After being washed in 0.1 M phosphate buffer for three times, the tissues were fixed in 1% osmium tetroxide. Then samples were dehydrated by graded ethanol with the last dehydrated procedure in 90% acetone. All above procedures were made at 4 °C. After being embedded in Epon Araldite and fixed, ultrathin sections (50–60 nm) were cut using an LKB-I ultramicrotome (Lecia, Germany) and stained with 3% uranyl acetate and lead citrate. Images were captured with the CM-120 transmission electron microscope (Philip, Holland).

### RNA interference, plasmid transfection, and real-time quantitative PCR

The siRNAs against REGγ, PP2Acα, FoxO3a, and negative siRNA (siREGγ, siPP2Acα, siFoxO3a, and siNeg, respectively) were synthesized by GenePharma (Shanghai). The target sequences of these siRNAs (5ʹ-3ʹ) are as follows: human siREGγ1#: UCUGAAGGAACCAAUCUUA, siREGγ2#: CUCAUCAUAUCAGAGCUGA, siREGγ3#: GAAGGAAAGUGCUAGGUGU, siPP2Acα: GGCAGAUCUUCUGUCUACA, siFoxO3a: CAACCTGTCACTGCATAGT, and rat siREGγ: GCAGAAGACTTGGTGGCAA. All cells in this study were transfected with the siRNAs by using Lipofectamine™ RNAiMAX (Invitrogen) and with the respective plasmids by using Lipofectamine™ LTX reagent with PLUS™ reagent (Invitrogen), according to the manufacturer’s instructions. Total RNA was extracted using TRIzol reagent (Takara), and first-stand cDNA was synthesized using reverse transcriptase (Takara). RT-qPCR with SYBR Green (Takara) was performed to examine the relative mRNA levels of indicated genes. Sequences for real-time qPCR primers are shown in Table S4.

### Immunoblotting, immunoprecipitation, immunofluorescence, and cell fractionation assay

Immunoblotting, immunoprecipitation, and immunofluorescence were performed as described previously [12, 14], immunofluorescence was analyzed with a laser scanning confocal microscopy (Lecia, Wetzlar, Germany) and were measured using Image-Pro Plus 6.0 software, the percentage of nuclear and cytoplasmic localization was determined by counting 400 positive cells from three replicates, and cell fractionation assay were performed as described [17].

### Luciferase assay

The pGL3–SOD2 promoter Luc reporter gene construct was generated using a plasmid pGL3-basic (Promega, Madison, WI, USA) by proof-reading PCR, containing the luciferase gene and a human SOD2 promoter fragment as described [32] to evaluate the transcriptional activity of the SOD2 promoter. AC16 cells were transiently transfected with the reporter gene construct and the indicated plasmids or siRNAs. Next, the cells were collected and washed once with cold PBS, followed by lysis in a cell lysis buffer (Promega, Madison, WI, USA). After one freezing and thawing cycle, whole-cell lysates were centrifuged in a cold room (4 °C) at 12,000 rpm for 15 min, and the supernatant obtained was collected in a fresh tube. Next, 20 μL supernatant was added to equal amounts of luciferase assay substrate, and luminescence was detected as relative light units by using the LUMIstar OPTIMA reader (BMG Labtech, Offenburg, Germany). Each assay was repeated three times. Fold change in values is represented as a mean of three experiments.

### In vitro protein degradation assay

Purified recombinant REGγ protein and 20S proteasome were purchased. The substrate PP2Acα protein was generated by in vitro TNT® Transcription/Translation System. The target protein decay assay was subsequently performed by incubating substrate, 20S proteasome, and purified REGγ protein as described previously [14] with appropriate controls. The results were analyzed by immunoblotting.

### Intracellular ROS measurement

Production of ROS was evaluated by analyzing the fluorescence intensity that resulted from DHE (Invitrogen) staining. In brief, frozen mouse hearts were cut into 5 μm sections. Serial heart sections were stained with 5 μM DHE at 37 °C for 30 min and then measured by fluorescence microscopy. AC16 cells were stimulated with Ang II for 6 h, followed by being loaded with 5 μM DHE at 37 °C for 30 min and then measured by fluorescence microscopy.

### Data collection and statistical analysis

At least three independent experiments were performed for each cellular experimental group and at least five independent experiments for each animal group in all cases unless otherwise stated, and all data were presented as the mean ± SD (standard deviation). Statistical analysis was performed by using the two-tailed, paired Student’s *t* test or one-way ANOVA (≥3 groups). A *P* value of less than 0.05 was considered statistically significant (*P < 0.05, **P < 0.01, and ***P < 0.001).

## Results

### REGγ expression is upregulated in the hypertrophic heart

**Fig. 1:**
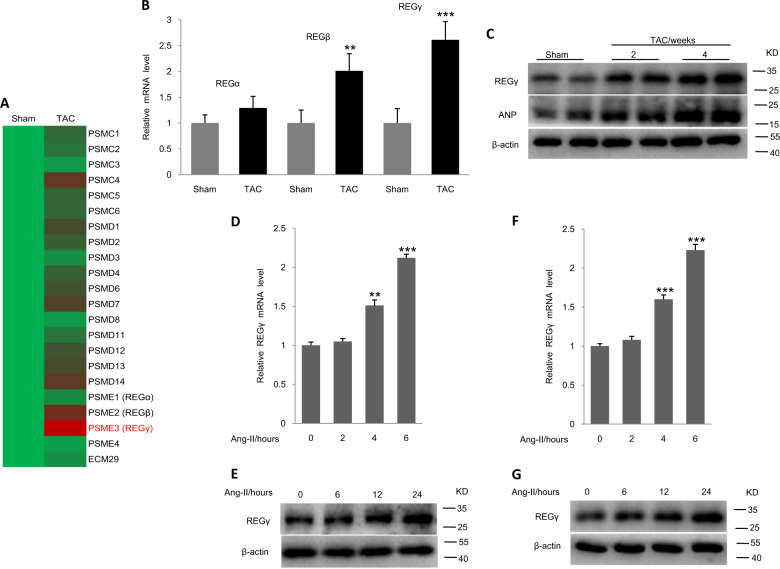
REGγ is increased in hypertrophic hearts. **a** Profile of the proteasome activators (subunits) expression in Sham or TAC-operated mouse heart at 4 weeks. **b** Relative fold change of REGα, REGβ, and REGγ mRNA expression in Sham or TAC-operated mouse heart at 4 weeks by RT-qPCR analysis (*n* = 6 per group, **P < 0.01, ***P < 0.001, Student’s *t* test). Protein levels of REGγ in mouse heart of Sham or TAC operation after 2 or 4 weeks (**c**) by immunoblotting analysis. mRNA and protein levels of REGγ in (**d**, **e**) NRCMs and (**f**, **g**) AC16 cells exposed to Ang II at different time points by RT-qPCR and immunoblotting analysis. (The experiments were repeated three times; error bars represent standard deviation, **P < 0.01, ***P < 0.001, Student’s *t* test).

To explore the proteasome activator (subunit) expression profile within the hypertrophic heart, we induced cardiac hypertrophy in wild-type (WT) mice by TAC operation and evaluated with RT-qPCR. We found that, among the 22 proteasome activators (subunits), REGγ (PSME3) was highly upregulated in the heart of TAC operation for 4 weeks (Fig. 1a). The relative increased fold of REGγ mRNA was shown (Fig. 1b) by RT-qPCR analysis. Meanwhile, the relative mRNA levels of REGβ (PSME2) was also increased but was less than that of REGγ (Fig. 1b). However, the expression of REGα (PSME1) was not different (Fig. 1b). The expression of REGγ was also upregulated in mouse heart in a time-dependent manner after TAC operation by immunoblotting (Fig. 1c) (A-type natriuretic peptide (ANP); a marker for cardiac hypertrophy). Moreover, the REGγ mRNA and protein levels were increased in Ang II-treated NRCMs (Fig. 1d, e) and AC16 cells (Fig. 1f, g) in a time-dependent manner. Overall, these results suggest that REGγ, one of the 11S proteasome activators, may play a critical role in the regulation of cardiac hypertrophy.

### REGγ deficiency ameliorates TAC-induced cardiac hypertrophy phenotypes

**Fig. 2:**
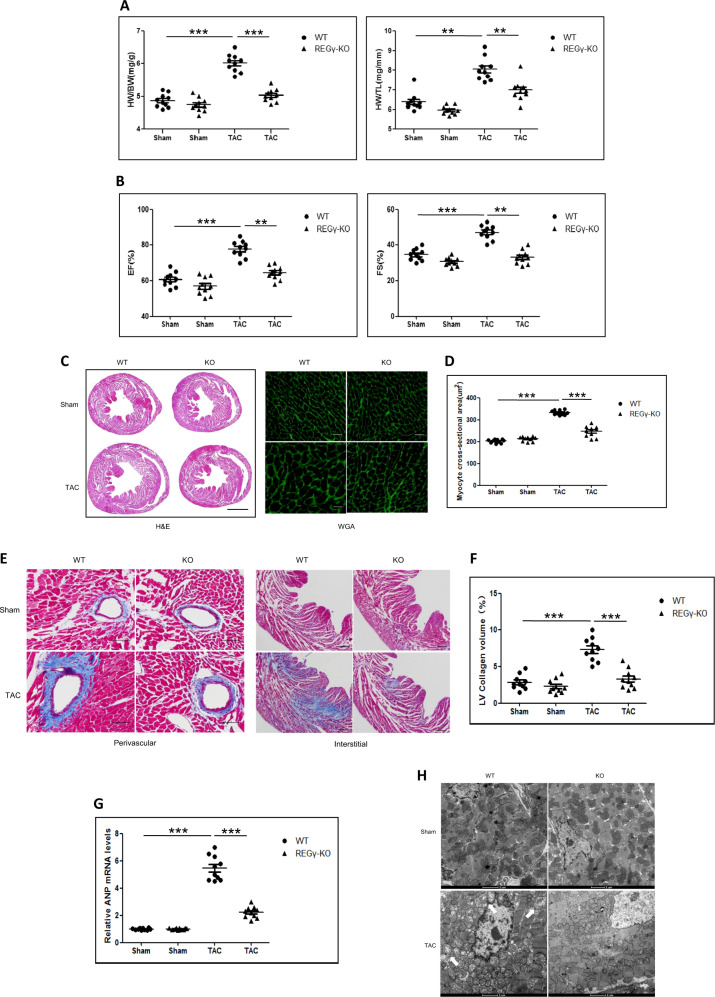
REGγ deficiency ameliorates TAC-induced cardiac hypertrophy phenotypes. **a** Heart weight to body weight (HW/BW) ratio and heart weight to tibia length (HW/TL) ratio. **b** Heart ejection fraction (EF) and fractional shortening (FS). **c** Heart haematoxylin and eosin (H&E) staining (scale bars: 2 mm). Wheat germ agglutinin staining (scale bars: 40 μm) and **d** corresponding quantitation graphs of myocyte cross-sectional area. **e** Masson staining to detect fibrosis of heart collagen (scale bars: 100 μm) and **f** corresponding quantitation graphs of heart fibrosis, and **g** heart ANP mRNA expression, and **h** representative transmission electron microscopy (TEM) images of the WT and REGγ-KO mice after sham or TAC operation for 4 weeks (*n* = 10 for each genotype; **P < 0.01, ***P < 0.001, one-way ANOVA test).

To study the association of REGγ deficiency with cardiac hypertrophy, we subjected REGγ global knockout mice (REGγ-KO) to TAC operation for 4 weeks. For the sham group, the heart weight to body weight (HW/BW) ratio of the REGγ-KO mice was indistinguishable from that of WT mice (Fig. 2a). However, TAC resulted in a dramatic increase in the HW/BW ratio in WT mice, whereas REGγ-KO mice showed no change (Fig. 2a). A similar effect was observed for the heart weight to tibia length (HW/TL) ratio (Fig. 2a). Furthermore, after 4 weeks of TAC, WT mice exhibited a compensatory increase in the ejection fraction and FS, whereas the sensitivity of cardiac performance to TAC operation was blunted by REGγ knockout (Fig. 2b). However, both the heart rates were indistinguishable between REGγ-KO and WT mice (Table S1). Histological analysis with H&E and WGA staining revealed that the cardiomyocyte hypertrophy induced by TAC operation was markedly ameliorated in the REGγ-KO mice (Fig. 2c, d). The evaluation of myocardial fibrotic areas by masson staining also revealed less fibrosis in the hearts of TAC operation-treated REGγ-KO mice compared with WT mice (Fig. 2e, f). Consistent with these data, REGγ deficiency significantly inhibited the TAC operation-induced upregulation of ANP mRNA levels (Fig. 2g). We also performed TEM to analyze the changes of mitochondrial morphology as pressure overload-induced cardiac hypertrophy is often associated with mitochondrial damage [33], more aggravated changes were observed in REGγ-WT mice compared with KO mice, characterized by much larger mitochondria, vacuolar changes, and disintegrate cristae (white arrows in Fig. 2h).These results demonstrate that REGγ deficiency protects against pathological cardiac hypertrophy. The characterization of REGγ-KO mice was detected by immunoblotting (Fig. S1a).

### REGγ deficiency improves cardiac oxidative stress in response to hypertrophic stimuli

**Fig. 3:**
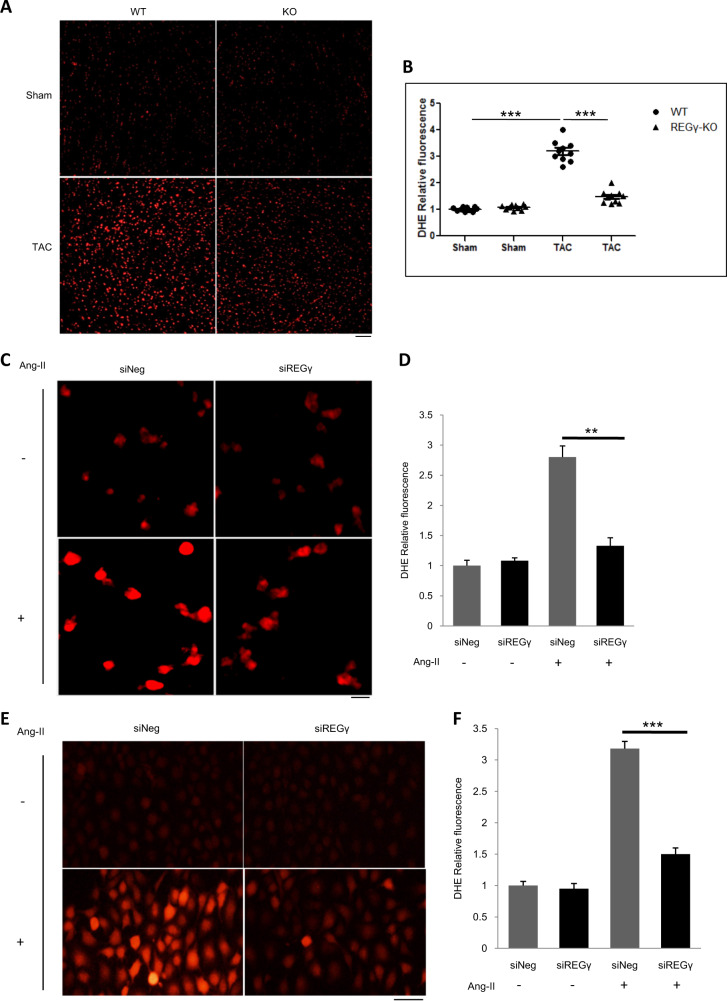
REGγ deficiency improves cardiac oxidative stress in response to hypertrophic stimuli. **a** REGγ deficiency inhibits cardiac ROS accumulation (scale bars: 50 μm) and **b** corresponding quantitation graphs of DHE relative fluorescence in mice after TAC operation for 4 weeks by DHE staining analysis (*n* = 10 for each genotype; *** P < 0.001, one-way ANOVA test). REGγ knockdown inhibits ROS accumulation (scale bars: 40 μm) and corresponding quantitation bar graphs of DHE relative fluorescence in (**c**, **d**) NRCMs and (**e**, **f**) human cardiomyocyte AC16 cells after Ang II treatment by DHE staining analysis. (The experiments were repeated three times; error bars represent standard deviation, **P < 0.01, ***P < 0.001, one-way ANOVA test).

Oxidative stress is well known to play a crucial role in the pathogenesis of cardiac hypertrophy and HF [34]. Hypertrophic stimuli such as pressure overload or Ang II increase cardiac ROS levels, and cardiac oxidative stress contributes to hypertrophic stimulus-induced cardiac hypertrophy or remodeling [35–37]. To this end, we assessed ROS levels in the hearts of REGγ-WT and KO mice. Interestingly, the results showed that TAC operation induced a dramatic increase in ROS levels (superoxide measured by DHE staining) in the hearts of WT mice, whereas this effect was significantly suppressed in REGγ-KO mice (Fig. 3a, b). REGγ knockdown also inhibited Ang II-induced ROS production in NRCMs (Fig. 3c, d) and human cardiomyocyte AC16 cells (Fig. 3e, f) in vitro. Collectively, these results indicated that REGγ was capable of increasing ROS levels during cardiac hypertrophy.

### REGγ interacts with PP2Acα and directs its degradation

To understand the molecular basis of REGγ-mediated regulation of oxidative stress during cardiac hypertrophy, we carried out mass spectrometry in mouse heart after TAC operation for 4 weeks, the most potential functional binding and degradable targets of REGγ (unique peptides ≥ 4) were evaluated and shown in Table S3. In the mass spectrometry and based on the oxidative stress and cardiac hypertrophy-related function and pathway analysis by previous reports and references [38–49], and the decrease of PP2Acα protein levels in mouse heart after TAC operation (Fig. S2a, b), lead us to explored and discovered that PP2Acα may be a potential target of REGγ in this process. The PP2A core enzyme, made up of 65-kDa scaffolding/A and a 36-kDa catalytic/C subunits, is regulated by the binding of one of many structurally distinct regulatory B subunits [38], and accounts for a significant percentage of all Ser/Thr phosphatase activity in most cells and tissues [39]. It also plays a crucial role in oxidative stress and cardiac hypertrophy regulation [40–49].

**Fig. 4:**
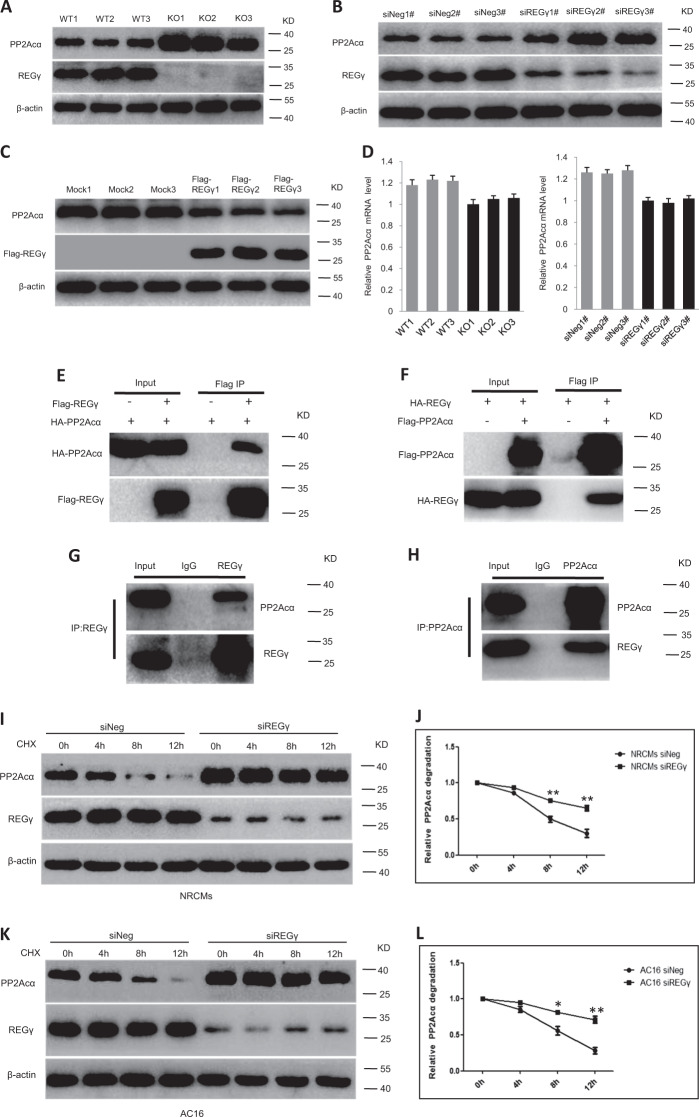

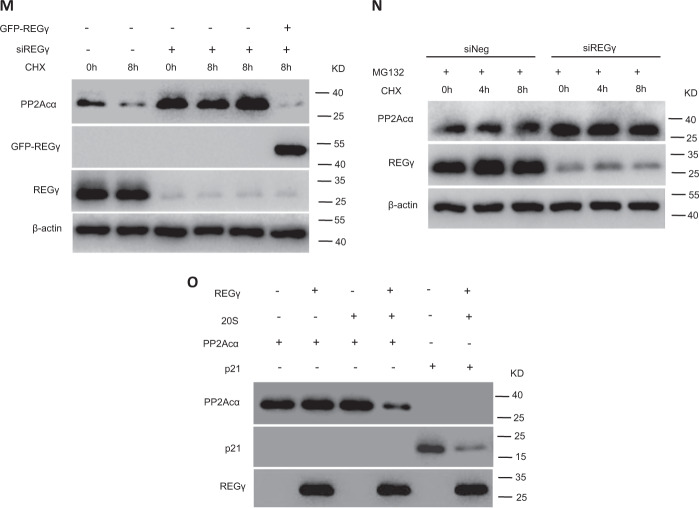
REGγ interacts with PP2Acα and directs its degradation. **a** REGγ knockout increases of PP2Acα protein levels in mice heart, and **b** REGγ knockdown upregulates PP2Acα protein levels, and **c** REGγ overexpression downregulates PP2Acα protein levels in AC16 cells by immunoblotting analysis, whereas **d** REGγ knockout or knockdown downregulates PP2Acα mRNA expression in murine heart or AC16 cells. **e** Interaction between REGγ and PP2Acα in 293T cells was determined by coimmunoprecipitation and immunoblotting analysis following transient transfection of Flag-REGγ/Flag vector and HA-PP2Acα into 293T cells. **f** Reciprocal interaction between REGγ and PP2Acα was performed by coimmunoprecipitation as indicated following transient transfection of Flag-PP2Acα/Flag vector and HA-REGγ into 293T cells. **g** Endogenous REGγ in AC16 cells was precipitated using anti-REGγ antibody or with IgG control, and coprecipitated PP2Acα was detected by immunoblotting. **h** Reciprocal interaction between endogenous REGγ and PP2Acα in AC16 cells was performed by using anti-PP2Acα antibody or IgG control, and coprecipitated REGγ was detected by immunoblotting. Stability of endogenous PP2Acα in (**i**) siNeg and siREGγ NRCMs and (**j**) corresponding quantitation graphs of relative PP2Acα degradation, or (**k**) siNeg and siREGγ AC16 cells and (**l**) corresponding quantitation graphs of relative PP2Acα degradation. (The experiments were repeated three times; error bars represent standard deviation, *P < 0.05, **P < 0.01, Student’s *t* test.) Cells were treated with CHX (100 μg/mL) for indicated times followed by immunoblotting. Stability of endogenous PP2Acα in (**m**) siNeg, siREGγ, and siREGγ plus GFP-REGγ plasmid AC16 cells and (**n**) AC16 cells pretreated with MG132. Cells were treated with CHX (100 μg/mL) for indicated times followed by immunoblotting. **o** REGγ directly promoted the degradation of PP2Acα. In vitro proteolytic analyses were performed using purified REGγ, 20S proteasome, and in vitro translated PP2Acα protein as indicated and described in “Materials and methods.” A known substrate of REGγ, p21, was shown as a positive control.

First PP2Acα protein and mRNA levels were examined by using mouse heart tissues or cell extracts from different cell types. The immunoblotting (Fig. 4a) results showed that PP2Acα protein levels were higher in REGγ-deficient heart tissues. Consistently, there was an increase in PP2Acα protein levels in AC16 cells after REGγ knockdown by several REGγ-specific siRNAs (siREGγ1#, 2#, and 3#) transfection, and decrease after REGγ overexpression by Flag-REGγ plasmid transfection (Fig. 4b, c). However, PP2Acα mRNA expression was lower in REGγ-deficient heart tissues or siREGγ AC16 cells compared with REGγ+/+ or siNeg (Fig. 4d), indicating that REGγ likely regulates PP2Acα by enhancing turnover of PP2Acα protein.

Following up, we confirmed the physical interaction between REGγ and PP2Acα by transiently expressing combinations of FLAG (a small peptide tag recognized by anti-Flag)-PP2Acα/HA-REGγ or HA-PP2Acα/FLAG-REGγ along with Flag vector control followed by reciprocal immunoprecipitation in 293T cells with FLAG-M2 agarose beads. The FLAG-tagged PP2Acα or REGγ successfully coimmunoprecipitated untagged REGγ or PP2Acα, whereas the Flag vector control failed to pull down proteins (Fig. 4e, f). To ensure the specificity, we also performed endogenous coimmunoprecipitation analysis by using AC16 cells lysates, and similar results (Fig. 4g, h) were observed.

To examined the activity of REGγ in PP2Acα protein degradation, we tested the degradation dynamics of PP2Acα. Following CHX treatment for various periods of time, PP2Acα was degraded faster in REGγ-WT NRCMs or AC16 cells than in REGγ-knockdown NRCMs or AC16 cells (Fig. 4i–l). We confirmed this in siNeg and siREGγ H9C2 cells (Fig. S3a, b). To ensure this, we performed the rescue experiment by REGγ pre-knockdown plus plasmid overexpression, as shown in Fig. 4m, PP2Acα was degraded dramatically in REGγ-WT AC16 cells, but not in REGγ knockdown, the REGγ-knockdown cells restore the function for PP2Acα degradation when transiently transfersed with the exogenous GFP-REGγ plasmid, indicated REGγ is required for PP2Acα protein stability. Moreover, we also performed proteasome inhibitor (MG132) treatment experiment to strengthen the role of REGγ-proteasome in PP2Acα degradation, as shown in Fig. 4n, when treated with MG132, the degradation of PP2Acα has no significant difference in REGγ-WT and REGγ-knockdown AC16 cells by following CHX treatment for various periods of time.

To ensure if the effect of REGγ on PP2Acα degradation is direct, we used cell-free proteolysis as described previously [14]. Incubation of in vitro translated PP2Acα with 20S proteasome or purified REGγ alone exhibited no significant degradation of PP2Acα, but a combination of REGγ and 20S proteasome promoted much faster turnover of PP2Acα than did the 20S proteasome alone (Fig. 4o). Therefore, we conclude that PP2Acα is a direct target of REGγ-proteasome in vitro and in cells.

### REGγ declines SOD2 expression

To determine the detailed pathway of REGγ regulated in this process, we examined the expression of some downstream direct key effectors in the regulation of oxidative stress during hypertrophic stimuli.

**Fig. 5:**
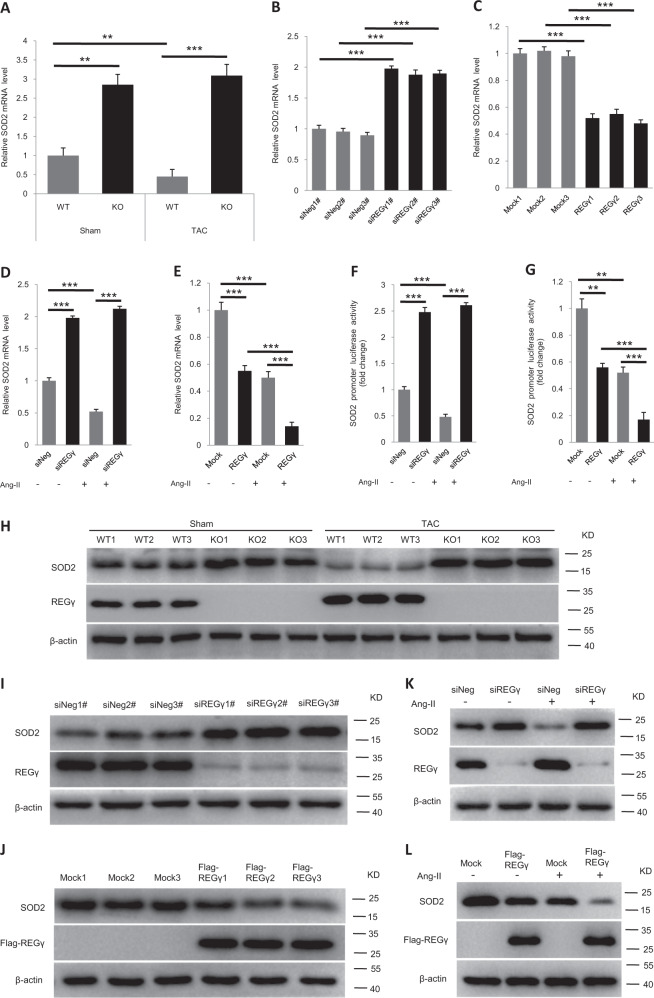
REGγ declines SOD2 expression. **a** REGγ deficiency inhibits the decline in cardiac SOD2 mRNA expression of mice in response to TAC operation for 4 weeks (*n* = 6 per group; **P < 0.01, ***P < 0.001, one-way ANOVA test). **b** REGγ knockdown upregulates SOD2 mRNA expression, and **c** REGγ overexpression downregulates SOD2 mRNA expression in human cardiomyocyte AC16 cells. (The experiments were repeated three times; error bars represent standard deviation, **P < 0.01, ***P < 0.001, one-way ANOVA test.) **d** REGγ knockdown inhibits the decline in SOD2 mRNA expression, and **e** REGγ overexpression promotes the decline in SOD2 mRNA expression in human cardiomyocyte AC16 cells in response to Ang II treatment. **f**, **g** Similar results were observed in AC16 cells by SOD2 promoter luciferase assays. (The experiments were repeated three times; error bars represent standard deviation, **P < 0.01, ***P < 0.001, one-way ANOVA test.) Similar consistent results were also observed by immunoblotting (**h**) in mice heart of sham or TAC operation for 4 weeks, and immunoblotting (**i**–**l**) in AC16 cells at indicated treatment.

In the intracellular space, several antioxidative enzymes (e.g., SOD2, SOD1, catalase, thioredoxin reductase [TrxR]) are critical for determining ROS levels and maintaining cardiac function [34, 35, 50–54]. The mRNA expression (Fig. 5a–e) analysis by RT-qPCR showed that REGγ knockout or knockdown (by several REGγ-specific siRNAs, siREGγ1#, 2#, and 3# transfection) significantly inhibited the decline in SOD2 expression induced by TAC in mouse hearts or by Ang II in AC16 cells, respectively, whereas REGγ overexpression (by Flag-REGγ plasmid transfection) had the opposite effect in AC16 cells. Similar results were observed in AC16 cells by SOD2 promoter luciferase assays (Fig. 5f, g). However, REGγ was unable to affect the expression of SOD1, catalase, or TrxR in sham or in the presence of TAC operation in mouse hearts (Fig. S4a–e, Fig. 5h–l). The immunoblotting results of SOD2 expression in mouse heart or AC16 cells after the same indicated treatment as the mRNA groups subjected were consistent with the mRNA and luciferase results showed. These findings indicate that REGγ predominantly inhibits the expression of SOD2 in the regulation of oxidative stress and cardiac hypertrophic stimuli. REGγ knockdown, and overexpression efficiency were quantified by real-time-qPCR assay (Fig. S5a–d).

### REGγ declines SOD2 expression in a PP2Acα–FoxO3a-dependent manner

We hypothesized that the PP2Acα pathway might be required for REGγ-dependent regulation of SOD2 gene expression in hypertrophic stimuli. It is known that FoxO3a is a key regulator of cell survival and reactive oxygen metabolism [55, 56] and can regulate SOD2 expression in response to oxidative stress in noncardiac cells [55]. This transcription factor is regulated by reversible phosphorylation and subcellular localization, is deactivated by kinase-directed phosphorylation, and activated by phosphatase-mediated dephosphorylation [57, 58]. Kinases such as Akt and serum- and glucocorticoid-induced kinase phosphorylate FoxO3a at the same sites, Thr32, Ser253, and Ser315 [59, 60], albeit with different affinities. There is cooperativity between these sites such that identification of Thr32 phosphorylation can be used to indicate phosphorylation of the other two sites and its activity [60, 61]. Reactivation of phosphorylated FoxO3a probably occurs via the ubiquitous threonine/serine phosphatase, protein phosphatase 2A [58], and may play role in different disorders progression [62–64].

**Fig. 6:**
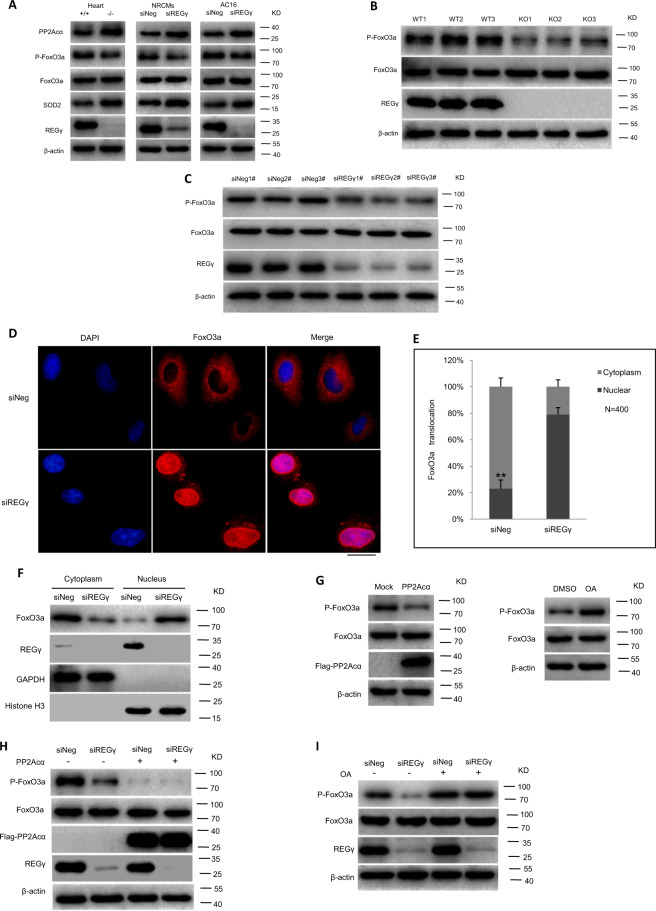

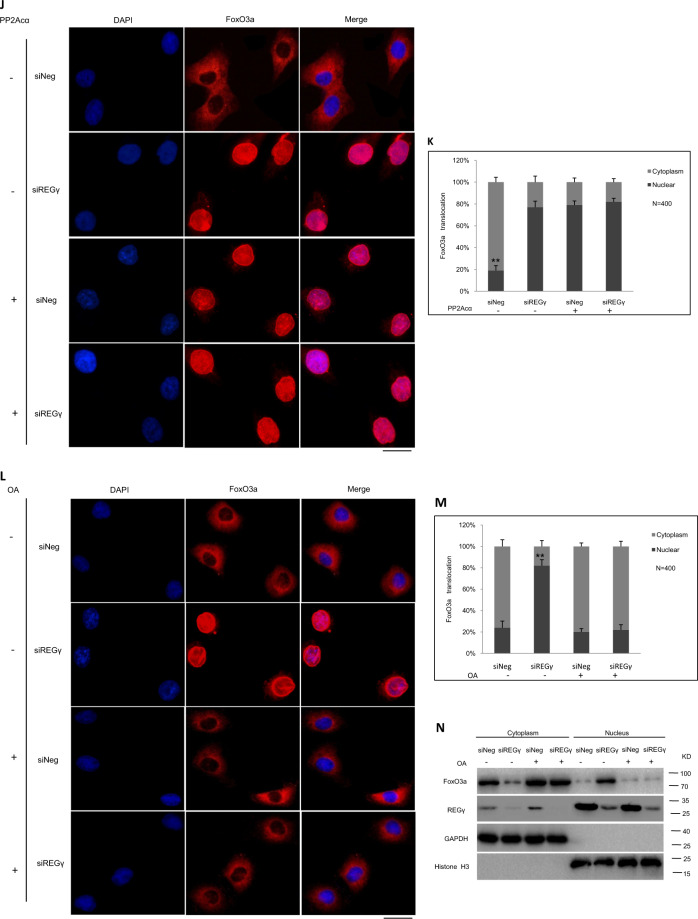

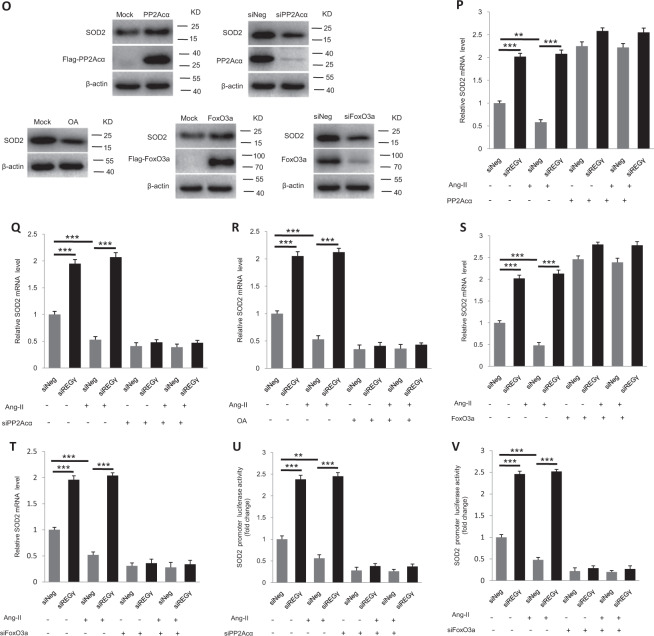
REGγ declines SOD2 expression in a PP2Acα–FoxO3a-dependent manner. **a** Protein levels of PP2Acα, P-FoxO3a, and SOD2 in REGγ+/+ and REGγ−/− heart tissue, and siNeg and siREGγ NRCMs and AC16 cells. Knocking out or silencing REGγ decreases FoxO3a phosphorylation levels in (**b**) murine heart tissue or in (**c**) human cardiomyocyte AC16 cells. REGγ knockdown promoted the translocation of FoxO3a from the cytoplasm to the nucleus in AC16 cells by **d** immunofluorescence analysis (scale bars: 20 μm) and **e** corresponding quantitation graphs, and **f** cell fractionation assay. (The experiments were repeated three times. Error bars represent standard deviation, **P < 0.01, Student’s *t* test.) **g** The levels of P-FoxO3a after OA treatment or PP2Acα overexpression in AC16 cells. **h** Overexpressing PP2Acα or **i** blocking PP2Acα activity by OA treatment significantly diminished the change of FoxO3a phosphorylation levels which were caused by REGγ knockdown in AC16 cells, similar results of (**j**–**m**) immunofluorescence analysis (scale bars: 20 μm) and corresponding quantitation graphs of FoxO3a translocation, and **n** cell fractionation assay was observed. (The experiments were repeated three times. Error bars represent standard deviation, **P < 0.01, one-way ANOVA test.) **o** Expression of SOD2 after PP2Acα overexpression, knockdown or activity inhibition by PP2Acα plasmid, siPP2Acα transfection or OA treatment, or FoxO3a overexpression or knockdown by FoxO3a or siFoxO3a plasmid transfection. **p**–**r** PP2Acα knockdown, overexpression, or activity inhibition dramatically rescued or attenuated SOD2 mRNA expression of Ang II stimuli regardless of REGγ levels. **s**, **t** FoxO3a overexpression or knockdown diminished the change of SOD2 mRNA expression of Ang II stimuli which REGγ caused. **u**, **v** Similar results of luciferase assays were observed in AC16 cells. (The experiments were repeated three times; error bars represent standard deviation, **P < 0.01, ***P < 0.001, one-way ANOVA test).

To evaluate the REGγ–PP2Acα–FoxO3a axis in SOD2 expression, we first tested protein levels of PP2Acα, P-FoxO3a (Thr32), and SOD2 together in REGγ+/+ and REGγ−/− heart tissue, and siNeg and siREGγ NRCMs and AC16 cells (Fig. 6a) by immunoblotting. Knocking out or silencing REGγ prevented decay of PP2Acα, a massive decrease of FoxO3a phosphorylation, and increase of SOD2 expression in REGγ-knockout cardiac tissues, or REGγ-knockdown cardiac cells and was associated with accumulated PP2Acα protein levels, suggested REGγ may via PP2Acα regulated FoxO3a phosphorylation and its downstream target SOD2 expression. To determine whether REGγ via PP2Acα regulated FoxO3a phosphorylation, we evaluated phosphorylation in REGγ+/+ and REGγ−/− heart tissue (Fig. 6b), and in AC16 cells after REGγ knockdown by several REGγ-specific siRNAs (siREGγ1#, 2#, and 3#) transfection (Fig. 6c). Knocking out or silencing REGγ decreased FoxO3a phosphorylation. To determine whether REGγ regulated the subcellular localization of FoxO3a followed by its phosphorylation, we performed immunofluorescence by using AC16 cells. We observed that REGγ knockdown by siREGγ transfection promoted the translocation of FoxO3a from the cytoplasm to the nucleus in AC16 cells (Fig. 6d, e). Similar results (Fig. S6a) were observed in NRCMs. Similar results of cell fractionation assays (Fig. 6f) were observed, REGγ knockdown promoted the nuclear localization of FoxO3a. Since PP2Acα as a FoxO3a binding partner and plays a critically direct role in the regulation of FoxO3a dephosphorylation, nuclear localization, and transcriptional activation is dependent upon their interaction [62]. First we confirmed their interaction here by endogenous reciprocal immunoprecipitation in AC16 cells (Fig. S7a, b), and with a cell-permeable inhibitor of PP2Acα (OA) treatment, endogenous P-FoxO3a accumulated, whereas PP2Acα overexpression decreased the endogenous P-FoxO3a levels in AC16 cells (Fig. 6g), substantiating negative regulation of FoxO3a phosphorylation by PP2Acα. Then to determine the causal relationships among REGγ, PP2Acα, and FoxO3a, REGγ-mediated regulation of FoxO3a phosphorylation was further analyzed before and after treatment with OA or PP2Acα plasmid transfection. Blocking PP2Acα activity by OA treatment or overexpressing PP2Acα by plasmid transfection significantly diminished the change of FoxO3a phosphorylation levels which REGγ knockdown caused in AC16 cells (Fig. 6h, i), indicating REGγ regulates FoxO3a phosphorylation in a PP2Acα-dependent manner. Similar results of (Fig. 6j–m) immunofluorescence and (Fig. 6n) cell fractionation assay were observed, indicating REGγ regulates FoxO3a intracellular localization in a PP2Acα-dependent manner. Taken together, our results demonstrate REGγ regulates FoxO3a phosphorylation and cellular localization in a PP2Acα-dependent manner.

Then we determined whether REGγ-mediated regulation of SOD2 expression could be affected by manipulation of PP2Acα levels and PP2Acα–FoxO3a axis (Fig. 6o). We first overexpressed PP2Acα or FoxO3a by exogenous plasmids, or knocked down PP2Acα or FoxO3a by RNA interference against PP2Acα or FoxO3a, or treated with PP2Acα–FoxO3a axis inhibitor (OA) to block the pathway respectively, and demonstrated that SOD2 indeed requires PP2Acα or FoxO3a for expression in AC16 cells. Then both REGγ knockdown and PP2Acα overexpression, knockdown, or inhibition, and FoxO3a overexpression or knockdown were simultaneously performed to determine whether REGγ regulates SOD2 expression in a PP2Acα–FoxO3a-dependent manner. Knockdown of REGγ significantly inhibited the decline in SOD2 mRNA expression with Ang II stimuli, but this REGγ-derived decrease was not observed when cells were overexpressed PP2Acα (Fig. 6p), knocked down PP2Acα (Fig. 6q) or treated with PP2Acα inhibitor OA (Fig. 6r), the similar results were observed when overexpressed FoxO3a (Fig. 6s) or knocked down FoxO3a (Fig. 6t) with real-time-qPCR detection in AC16 cells, the similar effects were observed for the SOD2 promoter luciferase assays when both knocked down REGγ and PP2Acα or FoxO3a (Fig. 6u, v). Taken together, our results demonstrate that REGγ regulates SOD2 expression in a PP2Acα–FoxO3a axis-dependent manner in response to hypertrophic stimuli, and may de on it in the regulation of oxidative stress and cardiac hypertrophy. REGγ knockdown, PP2Acα knockdown, and overexpression efficiency (Fig. S8a–e) and REGγ knockdown, FoxO3a knockdown, and overexpression efficiency (Fig. S9a–d) were quantified by real-time-qPCR assay.

### REGγ-induced cardiac ROS production and hypertrophy-related anomalies depend on PP2Acα–SOD2 axis

**Fig. 7:**
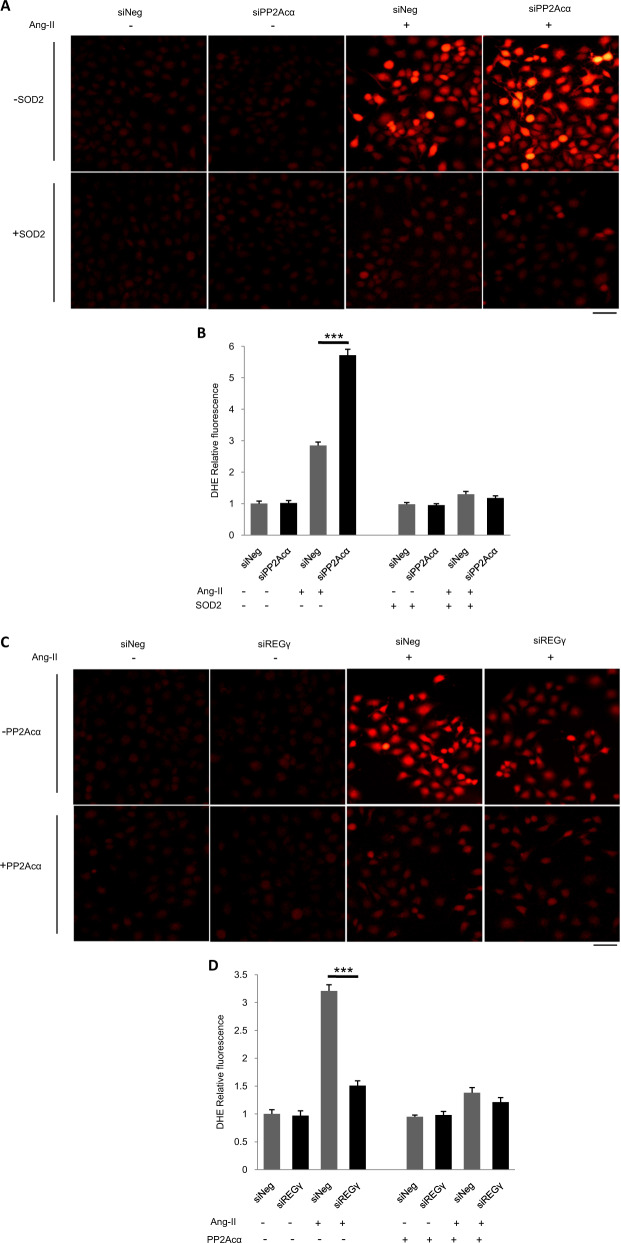

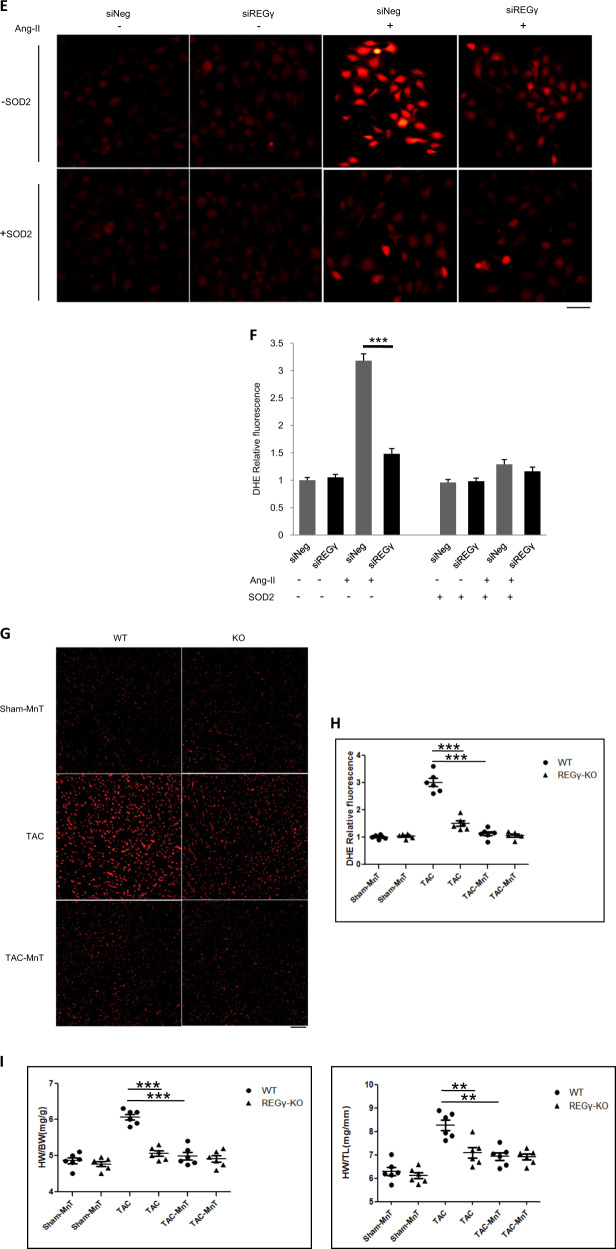

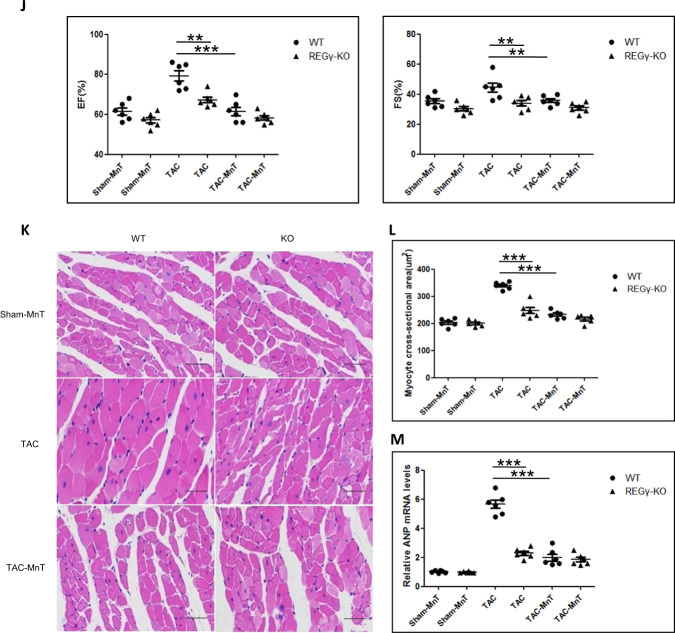
REGγ-induced cardiac ROS production and hypertrophy-related anomalies depend on PP2Acα–SOD2 axis. PP2Acα indeed requires SOD2 for the inhibition ROS accumulation in cardiomyocyte. **a**, **b** SOD2 overexpression inhibited ROS accumulation caused by PP2Acα knockdown, and **c**, **d** PP2Acα or **e**, **f** SOD2 overexpression rescued ROS accumulation caused by REGγ in Ang II-treated human cardiomyocyte AC16 cells by DHE staining (scale bars: 40 μm) and corresponding quantitation graphs of DHE relative fluorescence, indicating PP2Acα–SOD2 axis essentially contributes to the effects of REGγ on cardiac ROS accumulation. (The experiments were repeated three times; error bars represent standard deviation, ***P < 0.01, one-way ANOVA test.) MnTBAP (MnT) treatment prevented cardiac ROS production and hypertrophy-related anomalies that caused by REGγ. **g** Heart DHE staining (scale bars: 50 μm) and **h** corresponding quantitation graphs of DHE relative fluorescence, **i** heart weight to body weight (HW/BW) ratio and heart weight to tibia length (HW/TL) ratio, **j** heart ejection fraction (EF) and fractional shortening (FS), **k** heart haematoxylin and eosin (H&E) staining (scale bars: 40 μm), and (**l**) corresponding quantitation graphs of myocyte cross-sectional area, **m** heart ANP mRNA expression of the WT and REGγ-KO mice after indicated operation (Sham-MnT, TAC, or TAC-MnT) for 4 weeks (*n* = 6 for each genotype; ***P < 0.001, **P < 0.01, one-way ANOVA test).

We then investigated whether PP2Acα–SOD2 axis essentially contributes to the effects of REGγ on cardiac ROS accumulation and hypertrophy. To evaluate this, we first tested ROS levels in siNeg and siPP2Acα Ang II-treated AC16 with the rescue of SOD2 overexpression or not, and demonstrated that PP2Acα indeed requires SOD2 for the inhibition ROS accumulation in AC16 cells (Fig. 7a, b). Then, we performed a rescue experiment by PP2Acα or SOD2 overexpression in AC16 cells (Fig. 7c, d). PP2Acα or SOD2 overexpression (Fig. 7e, f) inhibited ROS accumulation caused by REGγ in Ang II-treated AC16 cells. REGγ, PP2Acα knockdown and PP2Acα, SOD2 overexpression were evaluated by immunoblotting (Fig. S10a–c). Then to determine whether REGγ regulated cardiac hypertrophy in vivo indeed by inhibiting ROS accumulation that we had demonstrated. We also performed a rescue experiment using superoxide dismutase mimetic, manganese 5, 10,15, 20-tetrakis-(4-benzoic acid) porphyrin-MnTBAP (MnT) in mice (Fig. 7g, h). MnTBAP (MnT) treatment indeed prevented ROS production in the hearts of TAC-treated WT and REGγ-KO mice. Notably, the administration of MnTBAP to TAC-treated WT mice reversed the HW/BW ratios to the same extent as in REGγ-KO mice (Fig. 7i), the similar effect was observed for the HW/TL ratio (Fig. 7i). Furthermore, TAC-treated WT mice that received MnTBAP exhibited a similar cardiac function to REGγ-KO mice (Fig. 7j). However, both the heart rates were indistinguishable between indicated REGγ-KO and WT mice groups (Table S2).The effects of REGγ on the hypertrophic response, measured according to the cross-sectional area (Fig. 7k, l) and ANP expression (Fig. 7m), were completely abolished by MnTBAP. Collectively, these data indicate that enhanced oxidative stress caused by PP2Acα decay and subsequent SOD2 decline plays a key role in the REGγ-mediated prohypertrophic effect.

## Discussion

In this study, we find that REGγ is significantly upregulated in the TAC-induced hypertrophic hearts and we describe a mechanism responsible for REGγ-mediated regulation of cardiac hypertrophy. REGγ regulates this process via increases in the cardiac ROS accumulation by targeting PP2Acα for degradation directly and subsequent SOD2 decline. In response to hypertrophic stimuli, REGγ is significantly increased and targets PP2Acα for degradation, which leads to increase of FoxO3a phosphorylation and nuclear export, and subsequent cardiac SOD2 decline and ROS accumulation, thus results in cardiac hypertrophy.

ROS affect nearly all of the key features of cardiac maladaptation, including the hypertrophic response, contractile dysfunction, extracellular matrix remodeling, and arrhythmia [65–67]. Although this study does not rule out other possible mechanisms by which REGγ promotes hypertrophy, inhibition of oxidative stress by MnTBAP was sufficient to block the REGγ-mediated hypertrophic response, indicating that ROS accumulation play a major role in this process. In humans, mutations of mitochondrial antioxidants (e.g., SOD2, catalase, GPx, and TrxR) increase the risk for cardiovascular diseases [68–71]. SOD2 is essential for normal heart function and even a relatively slight reduction or mutation in SOD2 function can result in cardiac dysfunction [51, 67]. In addition, SOD2 deficiency in mouse causes dilated cardiomyopathy [65, 72], the protein levels of SOD2 are markedly decreased in murine hypertrophic hearts and human failing myocardia [73, 74]. Our data showed that REGγ knockout protect against TAC or Ang II-induced SOD2 decline by targeting PP2Acα for degradation, indicated REGγ maybe the key factor in the improvement of PP2Acα-oxidative stress induced pathological cardiac hypertrophy.

Although previous studies have reported that PP2A protects noncardiac cells against oxidative stress in vitro [38], and PP2A negatively regulates the hypertrophic response and cardiomyocyte specific deletion of PP2A causes cardiac hypertrophy [39, 40], suggests PP2A may play a protective role in the cardiac hypertrophy progression, the detailed mechanism of PP2Acα in cardiac oxidative stress and hypertrophy is unclear, here we demonstrates the key pathway that PP2Acα regulated in this process, and first find REGγ plays role in cardiac hypertrophy by targeting PP2Acα. And although previous reports have suggested PP2A stimulates Forkhead box protein O (FoxO) activity may interplay with Akt or other proteins [62, 75], and identified PP2A as a FoxO3a binding partner and plays a critical role in the regulation of FoxO3a dephosphorylation, nuclear localization, and transcriptional activation is dependent upon their interaction [62], and we have also confirmed their interaction here and demonstrated REGγ regulates FoxO3a phosphorylation and intracellular localization in a PP2Acα-dependent manner in cardiac cells, there maybe other kinases or mediators involved in this regulation as well and the detailed mechanisms of specific binding sites and/or mediators between FoxO3a and PP2Acα in this process are also needed to explore and strengthen in future study.

Overall, we identify REGγ as a novel regulator of cardiac hypertrophy and provides a novel mechanistic insight into the likely link between proteasome-oxidative stress and cardiac hypertrophy, which may have a major impact on the understanding and treatment of cardiac hypertrophy and HF, but the detailed mechanism of interaction and activity regulation between REGγ and 20S proteasome for cardiac hypertrophy, and how REGγ transfers the PP2Acα into 20S proteasome for degradation is not very clear, further experiments on the precise regulation of REGγ-PP2Acα/proteasome and human samples should be considered in the future study, it may provide more evidence and therapeutic approach of modulating proteasome activity for hypertrophic diseases.

## Supplementary information

Supplementary Information

Fig. S1

Fig. S2

Fig. S3

Fig. S4

Fig. S5

Fig. S6

Fig. S7

Fig. S8

Fig. S9

Fig. S10
