# Supplementary material for: The proteasome activator REGγ accelerates cardiac hypertrophy by declining PP2Acα–SOD2 pathway
Source: Cell Death Differ. 2020 May 18;27(10):2952–72. doi: 10.1038/s41418-020-0554-8 (PMC7494903; doi:10.1038/s41418-020-0554-8)
Supplement: Supplementary file 1 — Supplementary Information [file 41418_2020_554_MOESM1_ESM.doc]

**Supplementary Information for**

The proteasome activator REGγ accelerates cardiac hypertrophy by declining PP2Acα-SOD2 pathway

Yifan Xie^1,2,3^, Yang Gao^1,3^, Rifeng Gao^1,3^, Wenlong Yang^1,3^, Zheng Dong^1,3^, Robb E. Moses^4^, Aijun Sun^1,2,3,*^, Xiaotao Li^4,*^, Junbo Ge^1,2,3,*^

^1^ *Department of Cardiology, Zhongshan Hospital, Fudan University, 180 Fenglin Road, Shanghai 200032, China*

^2^*Institutes of Biomedical Science, Fudan University, 180 Fenglin Road, Shanghai 200032, China*

^3^ *Shanghai Institute of Cardiovascular Diseases, 180 Fenglin Road, Shanghai 200032, China*

^4^ *Department of Molecular and Cellular Biology, Baylor College of Medicine, One Baylor Plaza, Houston, Texas 77030, USA*

^*^To whom correspondence may be addressed. E-mail: [angelsunsh@163.com](mailto:angelsunsh@163.com) (A.S.), [xiaotaol@bcm.edu](mailto:xiaotaol@bcm.edu) (X.L.) or [junbog@outlook.com](mailto:junbog@outlook.com) (J.G.).

**This file includes:**

**Supplementary Figures**

Fig. S1. Characterization of REGγ-KO mice. (A) Immunoblotting showing REGγ expression in heart samples from WT and REGγ-KO mice on the C57BL/6 background.

Fig. S2. The protein levels of PP2Acα in mice heart after TAC operation. (A and B) PP2Acα protein level in mouse heart samples from sham or TAC operation for 4 weeks.

Fig. S3. REGγ can degrade PP2Acα. (A and B) Endogenous PP2Acα was degraded faster in H9C2 siNeg cells than in siREGγ cells.The cells were treated with CHX (100 ug/mL) for indicated times.

Fig. S4. The effect of REGγ on the expression of other antioxidative enzymes. mRNA expression of (A) SOD1, (B) Catalase, (C) TrxR1, (D) TrxR2 or (E) TrxR3 in REGγ+/+ and REGγ−/− mice heart tissues after sham or TAC operation were examined by real time-qPCR.

Fig. S5. The efficiency of REGγ-knockdown and -overexpression in transfected cells. mRNA expression of (A, B, C and D) REGγ in AC16 cells after indicated siRNA or plasmid transfection were quantified by real time-qPCR.

Fig. S6. The effect of REGγ on the cellular localization of FoxO3a . (A) Immunofluorescence staining of FoxO3a in REGγ siNeg and siREGγ NRCMs cells.

Fig. S7. Interaction of PP2Acα and FoxO3a. (A and B) Endogenous reciprocal immunoprecipitation showing PP2Acα interacts with FoxO3a in human cardiomyocyte AC16 cells.

Fig. S8. The efficiency of REGγ-knockdown, PP2Acα-knockdown and -overexpression in transfected cells. mRNA expression of (A, B and C) REGγ or (D and E) PP2Acα in AC16 cells after indicated siRNA or plasmid transfection were quantified by real time-qPCR.

Fig. S9. The efficiency of REGγ-knockdown, FoxO3a-knockdown and -overexpression in transfected cells. mRNA expression of (A and B) REGγ or (C and D) FoxO3a in AC16 cells after indicated siRNA or plasmid transfection were quantified by real time-qPCR.

Fig. S10. The efficiency of PP2Acα-knockdown and SOD2-overexpression, REGγ-knockdown and PP2Acα or SOD2-overexpression in transfected cells. Protein expression of (A) PP2Acα and SOD2, (B and C) REGγ and PP2Acα or SOD2 in AC16 cells after indicated siRNA and plasmid transfection were examined by immunoblotting.

**Supplementary Tables**

Table S1. Heart rate of C57BL/6 WT and REGγ-KO mice following sham or TAC operation for 4 weeks.

Table S2. Heart rate of C57BL/6 WT and REGγ-KO mice following MnTBAP or/and TAC operation for 4 weeks.

Table S3. The most potential functional binding targets (Unique Peptides≥4) of REG γ in mouse heart after TAC operation for 4 weeks by mass spectrometry assay.

Table S4. The primers for real-time qPCR.

**Table S1. Heart rate of C57BL/6 WT and REGγ-KO mice following sham or TAC operation for 4 weeks.**

| **Sham** | | | **TAC** | | |
| --- | --- | --- | --- | --- | --- |
| **Parameters** | **WT** | **REGγ-KO** | | **WT** | **REGγ-KO** |
| **N** | 10 | 10 | | 10 | 10 |
| **HR(bpm)** | 621±37 | 635±22 | | 609±79 | 646±41 |

All values are presented as the mean ± SD. WT: wild-type; KO: knockout; TAC: transverse aortic constriction; HR: heart rate.

**Table S2. Heart rate of C57BL/6 WT and REGγ-KO mice following MnTBAP or/and TAC operation for 4 weeks.**

| **Sham-MnTBAP** | | | **TAC** | | | **TAC-MnTBAP** | | |
| --- | --- | --- | --- | --- | --- | --- | --- | --- |
| **Parameter** | **WT** | **REGγ-KO** | | **WT** | **REGγ-KO** | | **WT** | **REGγ-KO** |
| **N** | 6 | 6 | | 6 | 6 | | 6 | 6 |
| **HR(bpm)** | 578±43 | 596±32 | | 626±46 | 605±76 | | 620±82 | 589±96 |

All values are presented as the mean ± SD.

**Table S3. The most potential functional binding targets (Unique Peptides≥4) of REG γ in mouse heart after TAC operation for 4 weeks by mass spectrometry assay.**

| **Accession** | **Protein names** | **Gene names** | **Sequence coverage (%)** | **Unique peptides** | **MW[kDa]** |
| --- | --- | --- | --- | --- | --- |
| P63017 | Heat shock cognate 71 kDa protein | HSPA8 | 29.88 | 8 | 70.85 |
| Q91WE2 | Protein FAM192A | FAM192A | 23.58 | 6 | 28.89 |
| P63330 | Serine/threonine-protein phosphatase 2A catalytic subunit alpha isoform | PPP2CA | 18.09 | 5 | 35.59 |
| Q8C6G8 | WD repeat-containing protein 26 | WDR26 | 14.45 | 5 | 72.08 |
| P69566 | Ran-binding protein 9 | RANBP9 | 11.79 | 4 | 77.80 |
| P62960 | Nuclease-sensitive element-binding protein 1 | YBX1 | 12.73 | 4 | 35.90 |

**Table S4. The primers for real-time qPCR**

| **Primer** | **Sequence (5'to3'）** |
| --- | --- |
| REGγ-F | AAGGTTGATTCTTTCAGGGAGC |
| REGγ-R | AGTGGATCTGAGTTAGGTCATGG |
| Mu SOD2-F | CAGACCTGCCTTACGACTATGG |
| Mu SOD2-R | CTCGGTGGCGTTGAGATTGTT |
| Hu SOD2-F | GGAAGCCATCAAACGTGACTT |
| Hu SOD2-R | CCCGTTCCTTATTGAAACCAAGC |
| Mu PP2AC-F | ATGGACGAGAAGTTGTTCACC |
| Mu PP2AC-R | CAGTGACTGGACATCGAACCT |
| Hu PP2AC-F | CAGAGCACTTGATCGCCTACA |
| Hu PP2AC-R | CATGGCACCAGTTATATCCCTC |
| Hu Foxo3a-F | TCACGCACCAATTCTAACGC |
| Hu Foxo3a-R | CACGGCTTGCTTACTGAAGG |
| 18S-F | CATTCGAACGTCTGCCCTATC |
| 18S-R | CCTGCTGCCTTCCTTGGA |
| Mu ANP-F | ACCTGCTAGACCACCTGGAG |
| Mu ANP-R | CCTTGGCTGTTATCTTCGGTACCGG |
| Mu SOD1-F | AACCAGTTGTGTTGTCAGGAC |
| Mu SOD1-R | CCACCATGTTTCTTAGAGTGAGG |
| Mu Catalase-F | AGCGACCAGATGAAGCAGTG |
| Mu Catalase-R | TCCGCTCTCTGTCAAAGTGTG |
| Mu TrxR1-F | CCCACTTGCCCCAACTGTT |
| Mu TrxR1-R | GGGAGTGTCTTGGAGGGAC |
| Mu TrxR2-F | AAACCAGGACTTTGAATCTGGAG |
| Mu TrxR2-R | GATGTGGGGAACAGAGGTAGC |
| Mu TrxR3-F | GGCAACAGGGTGATGATCTTC |
| Mu TrxR3-R | CTGGAAAGTTCGGTCACATCC |
| Mu α-tubulin-F | TACACCATTGGCAAGGAGATTGTTCCT |
| Mu α-tubulin-R | GGCTGGGTAAATGGAGAACT |
