# Supplementary figures and images for: The proteasome activator REGγ accelerates cardiac hypertrophy by declining PP2Acα–SOD2 pathway
Source: Cell Death Differ. 2020 May 18;27(10):2952–72. doi: 10.1038/s41418-020-0554-8 (PMC7494903; doi:10.1038/s41418-020-0554-8)

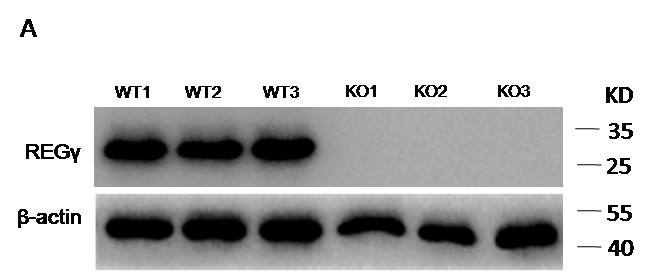

Supplement: Supplementary file 2 — Fig. S1 [file 41418_2020_554_MOESM2_ESM.tif]

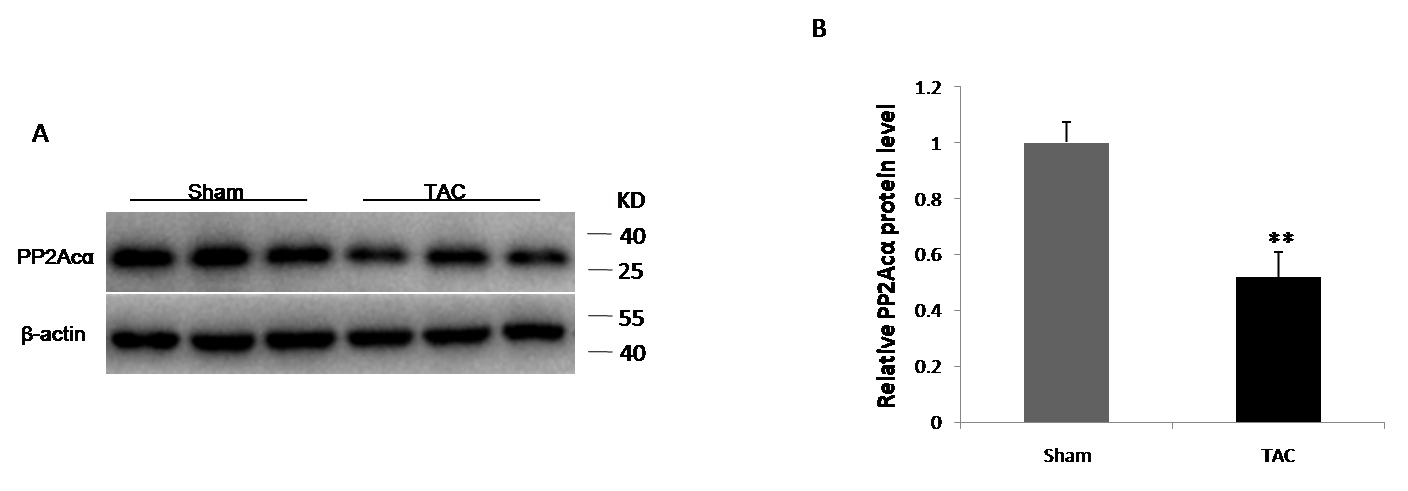

Supplement: Supplementary file 3 — Fig. S2 [file 41418_2020_554_MOESM3_ESM.tif]

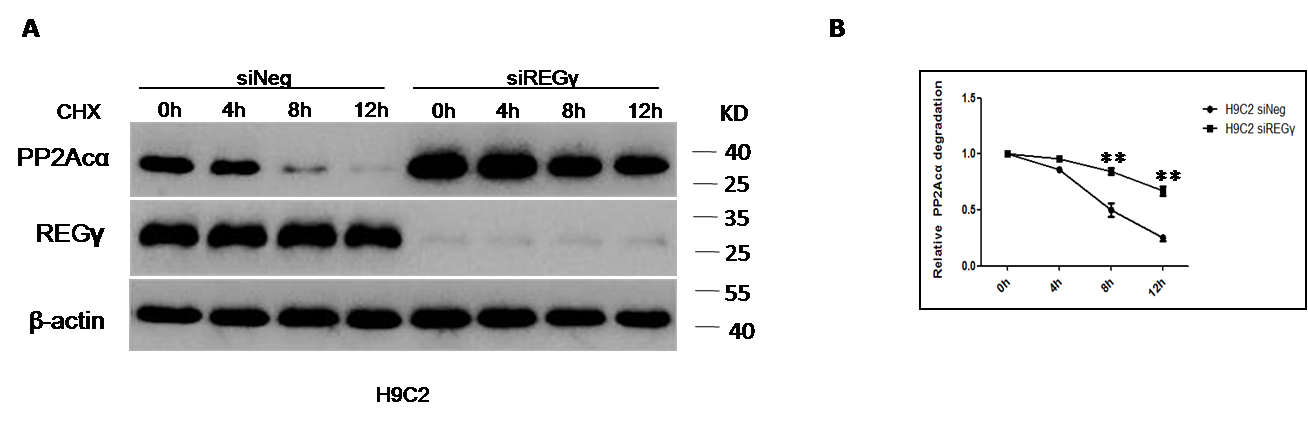

Supplement: Supplementary file 4 — Fig. S3 [file 41418_2020_554_MOESM4_ESM.tif]

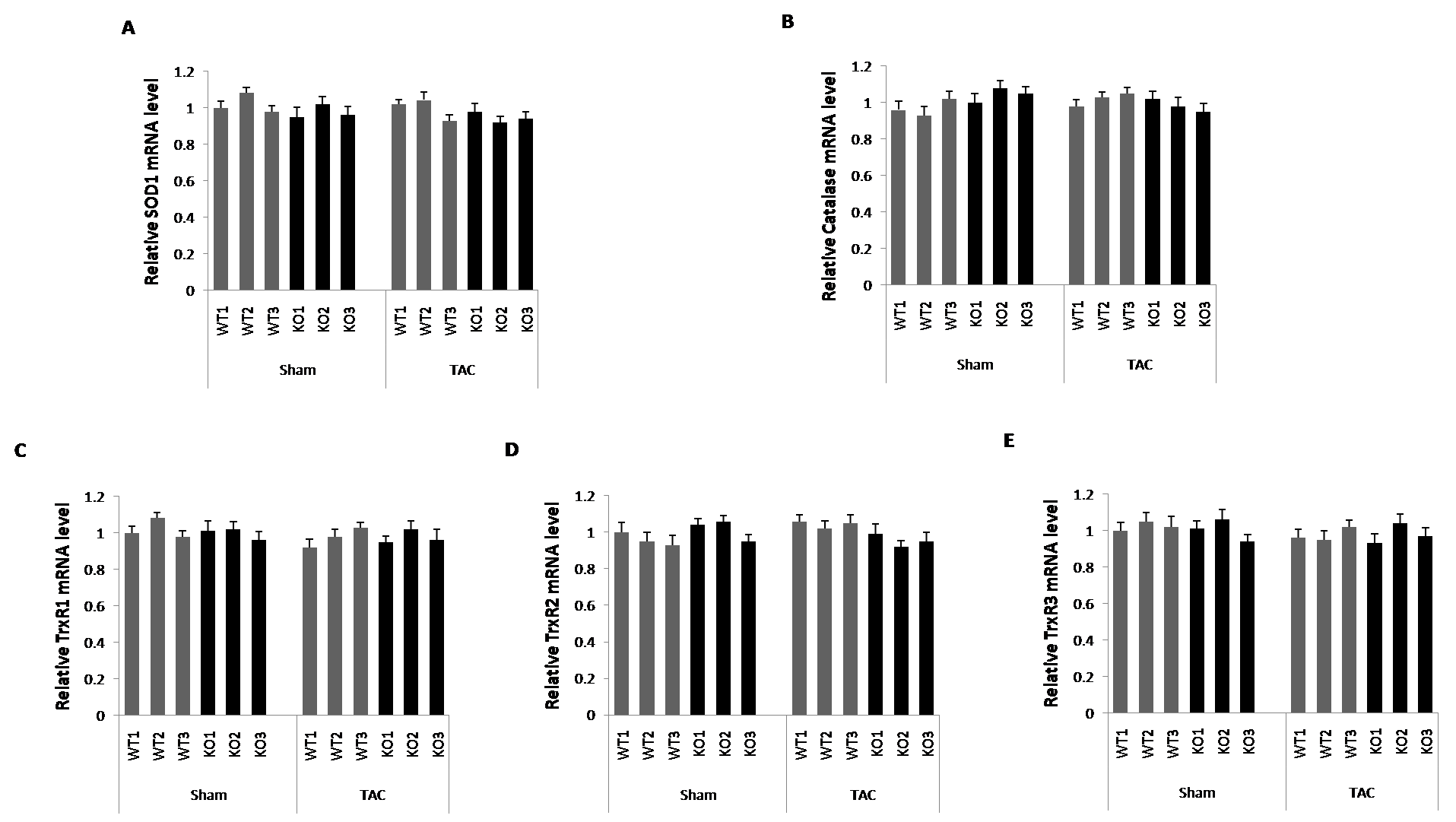

Supplement: Supplementary file 5 — Fig. S4 [file 41418_2020_554_MOESM5_ESM.tif]

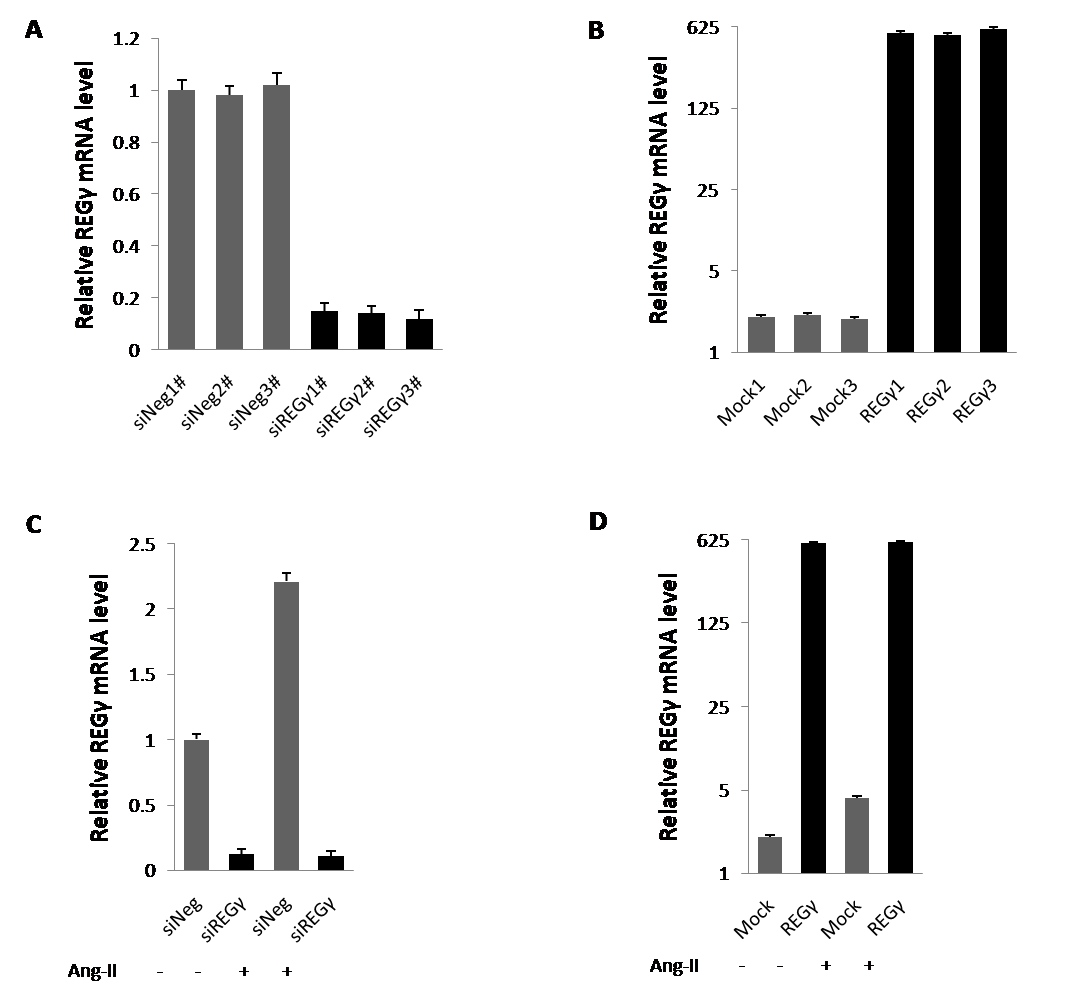

Supplement: Supplementary file 6 — Fig. S5 [file 41418_2020_554_MOESM6_ESM.tif]

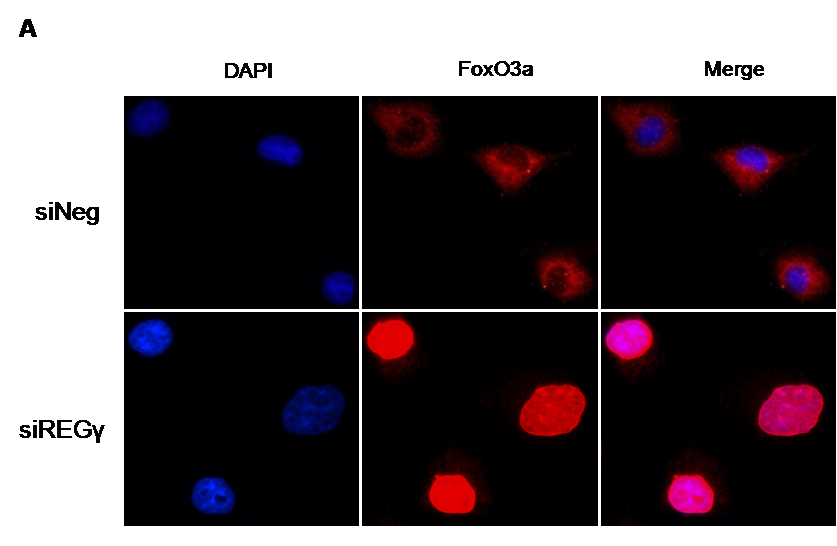

Supplement: Supplementary file 7 — Fig. S6 [file 41418_2020_554_MOESM7_ESM.tif]

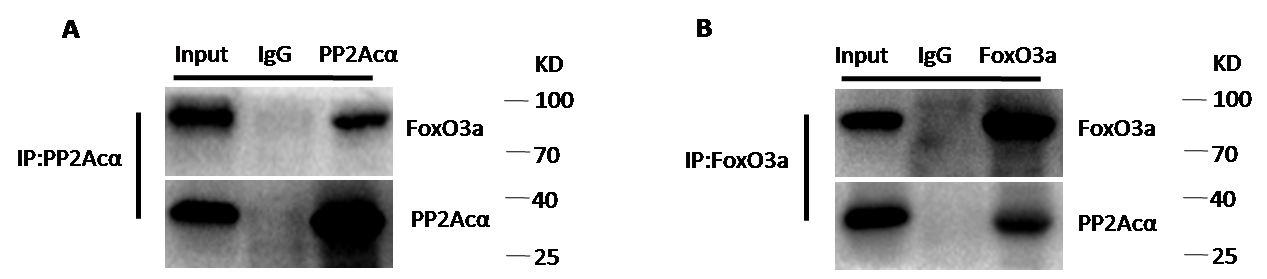

Supplement: Supplementary file 8 — Fig. S7 [file 41418_2020_554_MOESM8_ESM.tif]

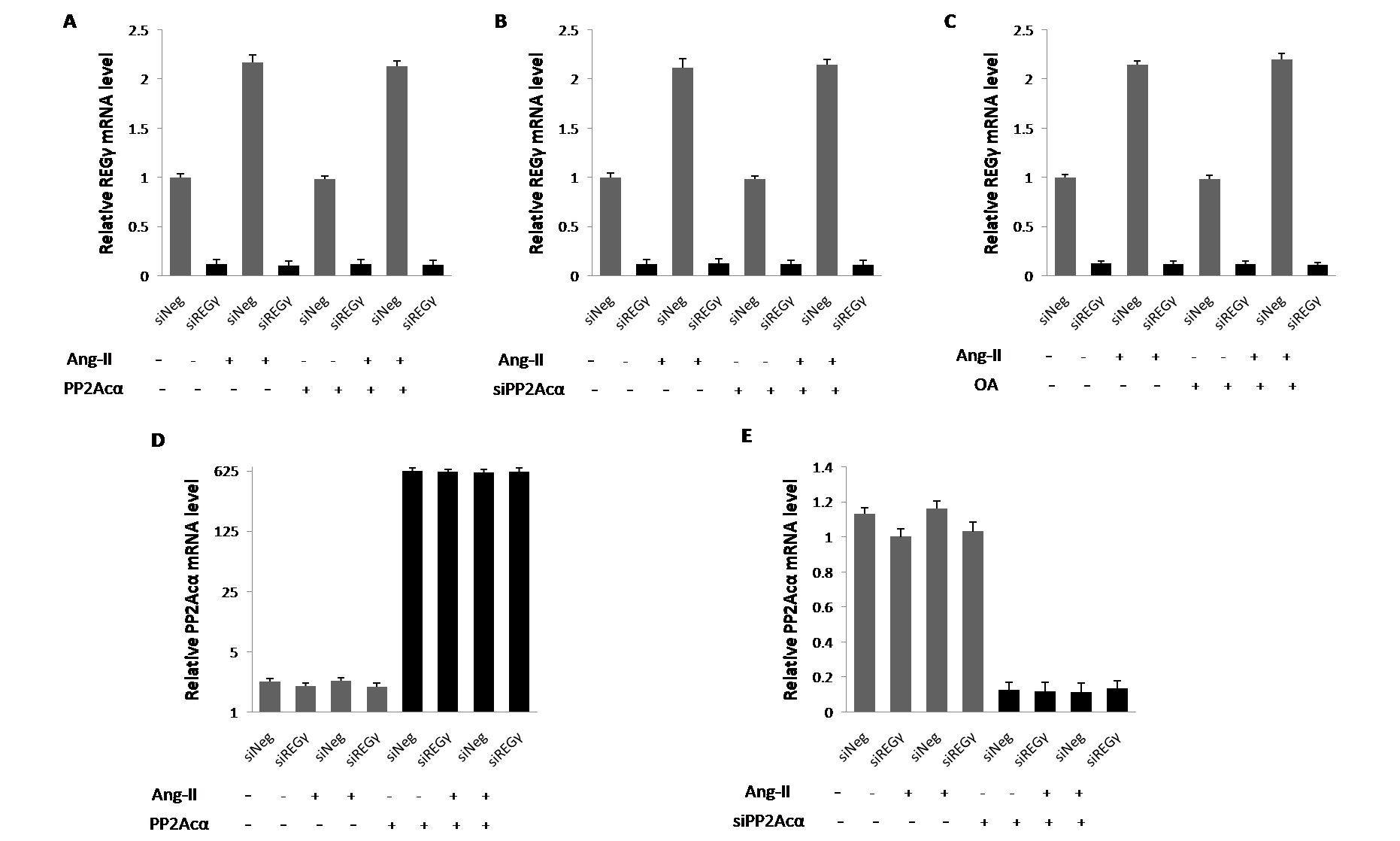

Supplement: Supplementary file 9 — Fig. S8 [file 41418_2020_554_MOESM9_ESM.tif]

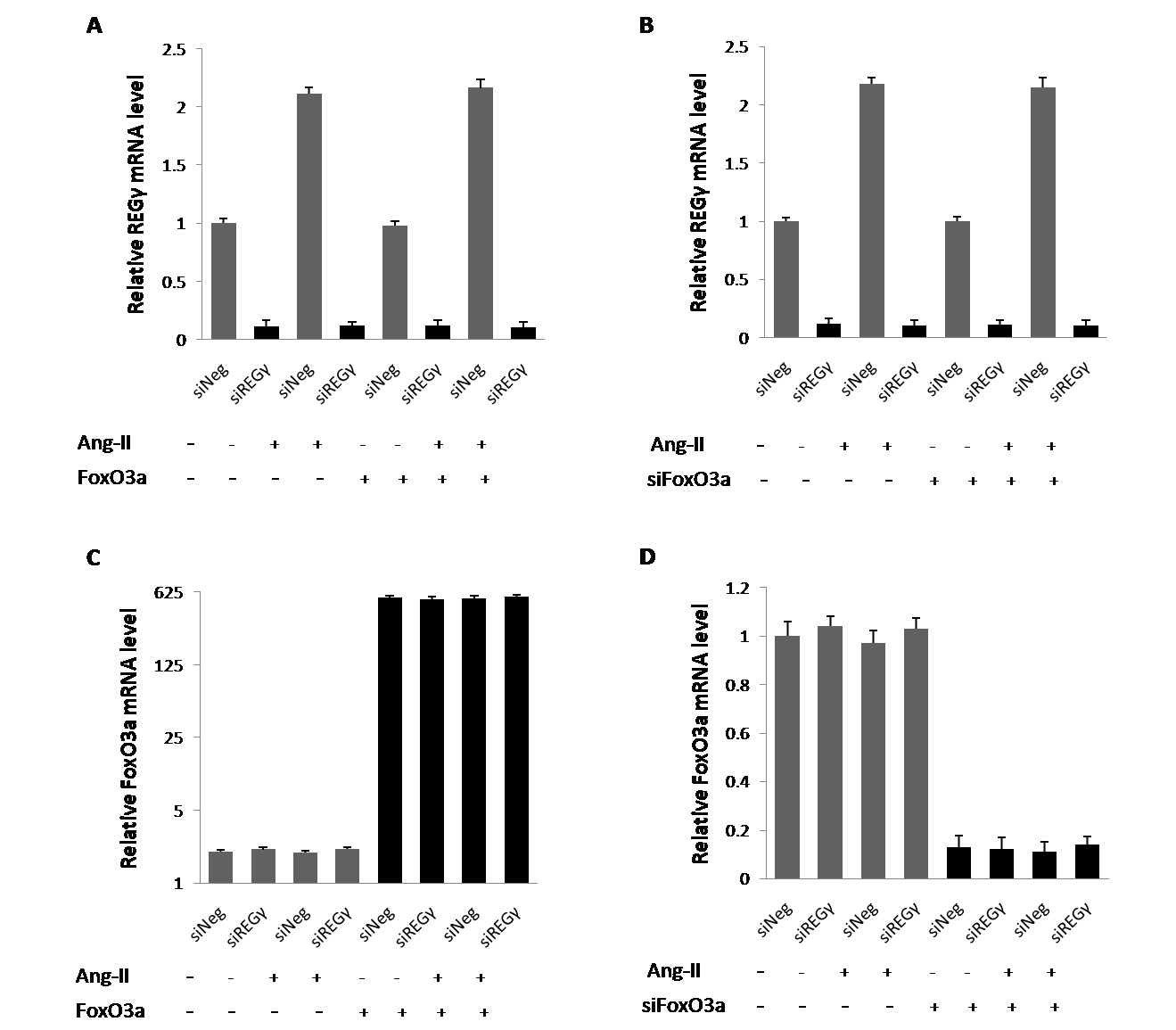

Supplement: Supplementary file 10 — Fig. S9 [file 41418_2020_554_MOESM10_ESM.tif]

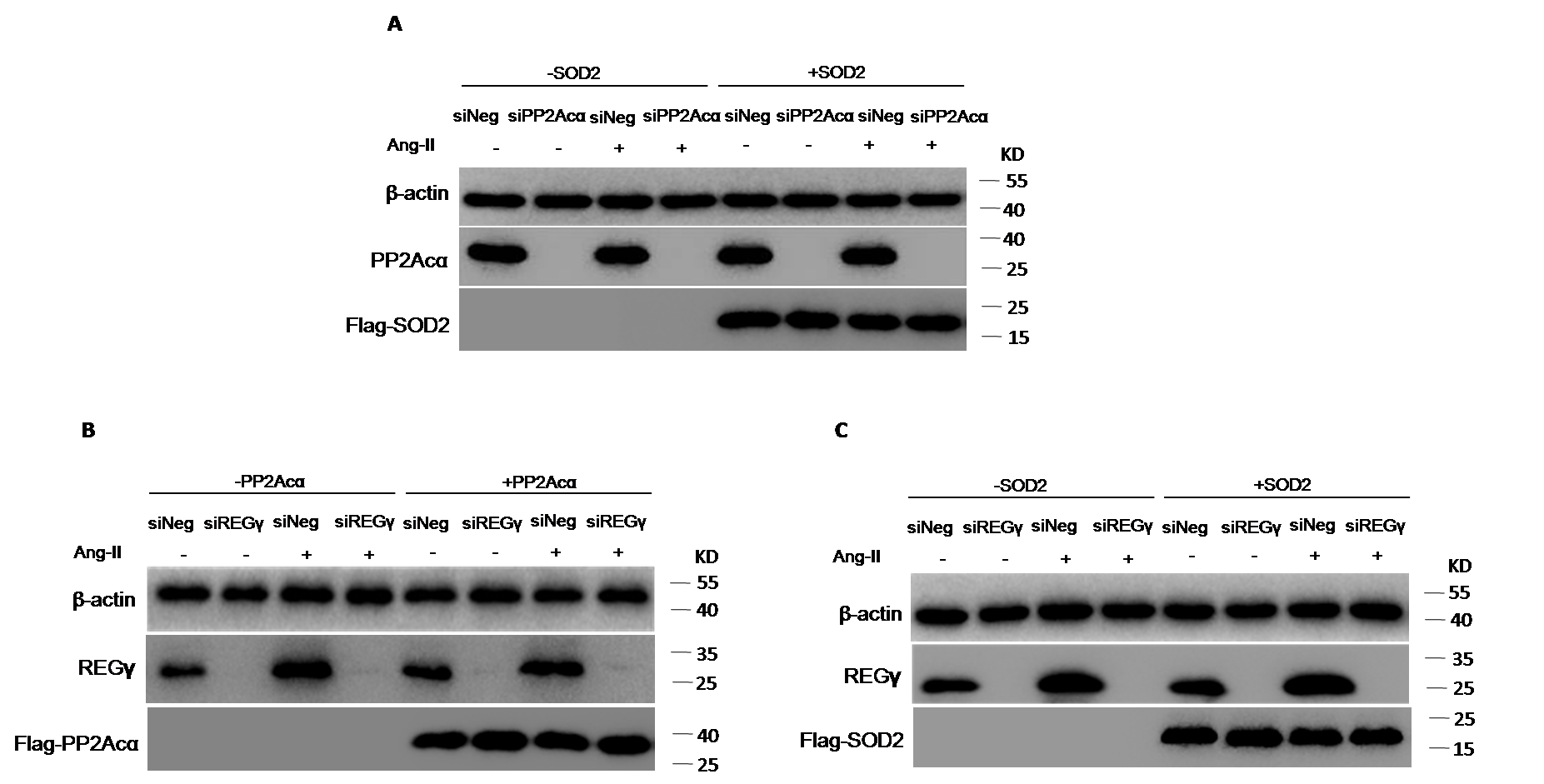

Supplement: Supplementary file 11 — Fig. S10 [file 41418_2020_554_MOESM11_ESM.tif]
